# Longitudinal Association of COVID-19 Hospitalization and Death with Online Search for Loss of Smell or Taste

**DOI:** 10.3201/eid2908.230071

**Published:** 2023-08

**Authors:** Derek Toomre, Sasikiran Kandula, Jeffrey Shaman

**Affiliations:** Yale University School of Medicine, New Haven, Connecticut, USA (D. Toomre);; Columbia University Mailman School of Public Health, New York, New York, USA (S. Kandula, J. Shaman);; Columbia University School of Climate, New York (J. Shaman)

**Keywords:** COVID-19, SARS-CoV-2, coronavirus disease, severe acute respiratory syndrome coronavirus 2, viruses, zoonoses, respiratory infections, surveillance, olfactory, Google Trends

## Abstract

Surveillance of COVID-19 is challenging but critical for mitigating disease, particularly if predictive of future disease burden. We report a robust multiyear lead-lag association between internet search activity for loss of smell or taste and COVID-19–associated hospitalization and deaths. These search data could help predict COVID-19 surges.

A challenge throughout the COVID-19 pandemic has been forecasting surges in hospitalizations and deaths so that health officials can plan and mitigate accordingly. However, effective COVID-19 surveillance and forecasting has been complicated by numerous factors: reported new cases variably underestimate true incidence; wastewater surveillance of SARS-CoV-2 is limited; variants have different virulence levels (*1*); and the risk for severe outcomes depends on previous immunizations, infections, and duration of the immune response, which is increasingly heterogeneous and variant-dependent. Ideally, independent proxies could help surveil the risk for increases in levels of severe COVID-19 disease; however, such proxies should be predictive and include a sufficient lead-lag relationship to enable public health mitigation. We investigated a possible lead-lag relationship between Google searches for “loss of smell” and “loss of taste” and COVID-19 hospitalizations and deaths.

Online search activity has previously been shown to have some predictive power for other diseases (*2*). Multiple symptoms are associated with COVID-19, but “new loss of smell or taste” is highly specific (odds ratio ≈10) (*3*). Loss of taste is confounded because flavor occurs partly through retronasal olfaction, and most persons do not differentiate between changes in taste versus flavor. In psychophysical smell and taste tests of persons with acute COVID-19, 72% had an olfactory defect and 19% had a gustatory defect (*4*). Early studies in the pandemic noted a correlation between Google Trends searches for loss of smell and taste and COVID-19 cases (*5*,*6*). This correlation occurred even before anosmia was publicly recognized as a COVID-19 symptom (*6*), underscoring the possibility that olfactory and gustatory symptoms are useful indicators for COVID-19 surveillance.

SARS-CoV-2–induced olfactory dysfunction has been studied at the cellular level and in human trials (*7*). Nasal sustentacular epithelial cells adjacent to olfactory neurons have high angiotensin-converting enzyme 2 receptor levels and are a key site of virus replication. SARS-CoV-2 enters cells either by fusing at the cell surface or in endosomes (*7*). Those 2 pathways vary between cell and tissue types; respiratory and olfactory epithelial cells use endosomal and cell surface pathways, and cell surface pathways require activation by cell surface proteases (e.g., TMPRSS2) (*7*). Mutations associated with Omicron caused it to be TMPRSS2-resistant (*8*) and display enhanced replication in the upper respiratory tract, consistent with less severe lung disease, lower mortality rates (*9*), and less frequent self-reported olfactory dysfunction (*10*). A hypothetical correlate is that olfactory dysfunction might be a proxy for general risk for infection of lung cells at the population level. Given this potential link, we examined whether Internet searches for “loss of smell” and “loss of taste” correlate with waves of COVID-19 deaths with a lead-lag relationship, and if so, whether that correlation is maintained across different waves of COVID-19 variants. 

To robustly test for a potential association, we analyzed Google Trends searches for “loss of smell” and “loss of taste” across 5 different English-speaking countries and 3 different years (2020, 2021, and 2022) and examined the correlation to reported COVID-19 hospitalizations and deaths (Figure). We retrieved weekly query frequencies for “loss of smell” (or “anosmia”) and “loss of taste” (or “ageusia”) from the Google Extended Trends API for Australia, Canada, South Africa, the United Kingdom, and the United States. Using public sources, we computed weekly COVID-19–associated mortality and hospitalization rates for February 2020–August 2022. For each country, we computed cross-correlation between paired search trend and outcome for each week between −6 (lead) and 6 (lag) for the study period and each calendar year (Appendix).

**Figure:**
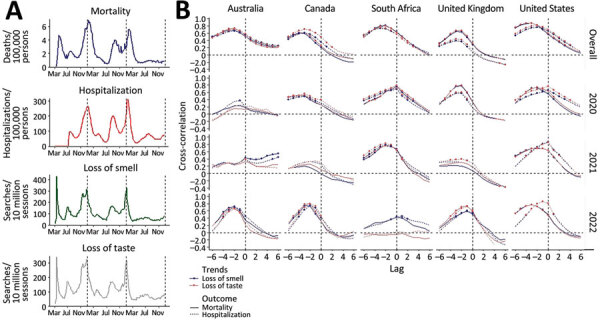
Longitudinal association of COVID-19 hospitalization and death with online search for loss of smell or taste. A) Weekly COVID-19–associated deaths (per 100,000 population), hospitalizations (per 100,000 population), and Google search trends for ‘loss of smell’ and ‘loss of taste’ (per 10 million search sessions) in the United States during March 2020–September 2022. Vertical broken lines delimit calendar years. B) Cross-correlation between Google trends of the 2 search queries, and the 2 outcomes in 5 countries (columns) over the entire COVID-19 pandemic period of March 2020–September 2022 (top row) and disaggregated by calendar year. Statistically significant correlations (p<0.01) are indicated by a data point. Lag between paired search trend and outcome is shown in weeks.

We observed a strong correlation in the United States between deaths, hospitalization, and searches for loss of smell or taste with surprisingly similar amplitudes for all major waves (Figure, panel A), including those associated with Omicron in December 2021. Cross-correlation was high (0.68–0.85) and significant (p<0.01) across all 5 countries; the peak trend for loss of smell or taste preceded hospitalization and deaths by 2–3 weeks (Figure, panel B). This correlation was seen across all years combined and was evident for most country–year combinations. The association appeared weak in years when outcome rates were low (e.g., Australia in 2020). The analysis indicates the correlation is robust over 3 years and multiple variant waves and that loss of smell or taste might give officials a useful lead indicator of the risk for COVID-19–associated hospitalizations and deaths. However, if this finding is to be used predictively, the persistence of this association would need to be closely tracked and monitored.

Strengths of this investigation are the long-duration longitudinal analysis across multiple countries, the use of simple search criteria and variable search terms, and analysis of the temporal lead-lag relationship. Limitations include potential for bias on the basis of media news cycles, the population scale of the analysis, and socioeconomic selection bias related to internet access. Future correlations will need to be monitored. Search activity might be a more useful indicator of infection levels than COVID-19–associated deaths. Despite these caveats, this accessible metric should be considered as a public health predictor.

Appendix Additional information about longitudinal association of COVID-19 hospitalization and death with online search for loss of smell or taste.
