# Supplementary material for: Longitudinal Association of COVID-19 Hospitalization and Death with Online Search for Loss of Smell or Taste
Source: Emerg Infect Dis. 2023 Aug;29(8):1711–3. doi: 10.3201/eid2908.230071 (PMC10370832; doi:10.3201/eid2908.230071)
Supplement: Appendix — Additional information about longitudinal association of COVID-19 hospitalization and death with online search for loss of smell or taste. [file 23-0071-Techapp-s1.pdf]

Article DOI: https://doi.org/10.3201/eid2908.230071

EID cannot ensure accessibility for supplementary materials supplied by authors. Readers who have difficulty accessing supplementary content should contact the authors for assistance.

Longitudinal Association of COVID-19 Hospitalization and Death with Online Search for Loss of Smell or Taste

Appendix

```
## Aggregate daily data to weekly resolutions
aggregateToWeek <- function(x){
  x <- merge(x, date.week.map, by = 'date')
  x <- x[, j = .(deaths = sum(deaths,na.rm = T),
              hosp = sum(hosp,na.rm = T),
              pop = mean(pop)),
        by = .(region, year, week)]

  x <- merge(x,
             date.week.map[day = = 1, j = .(date, year, week)],
             by = c('year', 'week'))

  x[,j = .(date,
        region,
        deaths = deaths*1E5/pop, # per 100000 population
        hosp = hosp*1E5/pop, # per 100000 population
        pop)]
}
```

```
## Function to find for correlations for outcomes vs trends in a county
doCountry <- function(req.region = 'United States', req.nrands = 10){
  x <- merge(d[region = = req.region and variable %in% c('smell'),
             j = .(date, smell = value)],
            mort[region = = req.region, j = .(date, deaths)],
            by = 'date')
  x <- merge(x, d[region = = req.region and variable %in% c('taste'),
               j = .(date, taste = value)],
            by = 'date')
  x <- merge(x, mort[region = = req.region, j = .(date, hosp)],
            by = 'date', all.x = T)


  smell <- cbind(x$date, scale(x$smell))
  taste <- cbind(x$date, scale(x$taste))
  mort <- cbind(x$date, scale(x$deaths))
  hosp <- cbind(x$date, scale(x$hosp))


  # Calculate cross-correlation for each pair and stack
  rbind(
    data.table(var1 = 'smell', var.2 = 'mort',
             pairSignalWrapper(smell, mort)),
    data.table(var1 = 'taste', var.2 = 'mort',
             pairSignalWrapper(taste, mort)),
    data.table(var1 = 'smell', var.2 = 'hosp',
             pairSignalWrapper(smell, hosp)),
    data.table(var1 = 'taste', var.2 = 'hosp',
             pairSignalWrapper(taste, hosp))
 )
}
```

```
### For the two time series, find correlations over the entire period and
### by calendar year
pairSignalWrapper <- function(x, y){
  overall <- pairSignal(x[,2], y[,2]) # over the entire study period
  y20 <- pairSignal(x[checkDate(x, 2020),2],
                    y[checkDate(y, 2020),2]) # calendar year 2020 only
  y21 <- pairSignal(x[checkDate(x, 2021),2],
                    y[checkDate(y, 2021),2]) # calendar year 2021 only
  y22 <- pairSignal(x[checkDate(x, 2022),2],
                    y[checkDate(y, 2022),2]) # calendar year 2022 only

  ret <- rbind(
    data.table(period = 'Overall', overall),
    data.table(period = '2020', y20),
    data.table(period = '2021', y21),
    data.table(period = '2022', y22))

  ret$period <- factor(ret$period,
                       levels = c('Overall', as.character(2020:2022)))

  ret
}


## Function to calculate cross-correlation between the two signals
pairSignal <- function(x, y){
  obj <- ccf(x, y, lag.max = 6, plot = F, type = 'correlation')
  p.value <- 2* (1 - pnorm(abs(obj$acf),
                           mean = 0,
```

```
                        sd = 1/sqrt(obj$n.used))) %>%
    round(4)
  ret <- data.frame(lag = −6:6, coeff = round(obj$acf, 4), p.value)

  ret
}
```

## Check if date is in a calendar year

```
checkDate <- function(x, req.year = 2020){
  year(as.Date(x[, 1], '1970–01–01')) =  = req.year
}
```

## Load mortality and hospitalization data from Our World in Data and
## aggregate daily data to week

```
<- readRDS(paste0(baseDir, 'owid-covid-data_v2.Rds'))
mort <- mort[date < = '2022–12–31',
            j = .(region = location, date, deaths = new_deaths, hosp = hosp_patients,
pop = population)] %>%
  aggregateToWeek()
```

## Load trends from Google Health Trends API

```
d <- readRDS(paste0(baseDir, 'Trends_v2.Rds'))
setnames(d, c('region', 'date', 'smell', 'taste'))
d$date <- as.Date(d$date)
d <- melt(d[date> = '2020–01–01' and date < = '2022–12–31'], id.vars = c('region', 'date'),
variable.factor = F)
```

## Stack datasets

```
temp <- rbind(mort[,.(variable = 'mort', region, date, value = deaths)],
          mort[,.(variable = 'hosp', region, date, value = hosp)], d, use.names = T)
temp$variable <- factor(temp$variable,
                levels = c('mort', 'hosp', 'smell', 'taste'),
                labels = c('Mortality', 'Hospitalization', 'Loss of smell', 'Loss of taste'))



### Calculate correlations
ret <- lapply(unique(temp$region),
          doCountry)

## post process to clean outcomes and trend labels
names(ret) <- unique(temp$region)
ccf.pairs <- rbindlist(ret, idcol = 'region')

ccf.pairs$var1 <- factor(ccf.pairs$var1,
                 levels = c('smell', 'taste'),
                 labels = c('loss of smell', 'loss of taste'))
ccf.pairs$var2 <- factor(ccf.pairs$var2,
                 levels = c('mort', 'hosp'),
                 labels = c('Mortality', 'Hospitalization'))
```
